# A Dental Efficiency Test

**Published:** 1916-07

**Authors:** Edward S. Barber

**Affiliations:** Chicago, Ill.


### A DENTAL EFFICIENCY TEST.

BY EDWARD S. BARBER, D.D.S., CHICAGO, ILL.

So much attention is given to the subject of efficiency nowadays, both in laity and professional magazines, that the time seems opportune to present the simple table herewith given, showing how any dentist can ''add himself up'' and determine whether or not to be satisfied with his present system of doing business.

It is a well established fact that there are two distinct sides to dentistry—the professional and the business sides. The first applies to the service we, as dentists, render to humanity, and the second applies to the means by which we support ourselves and families.

The professional side of our development is absolutely dependent, in the majority of cases, on the development of our business instinct, for good dentistry can not be done for a small fee, and most men do the kind that will earn a small fee quickly, not taking into consideration the subject of the patient's health in so doing.

A well known dentist told me he could make a better crown for $25.00 than for $10.00 If this is true, and I believe it is, the patient should be educated to pay more in order to get the best dentistry, and here is where business efficiency is of as much value to the patient as to the dentist who practices it.

If each man who reads this will privately add up his own percentages of efficiency, he will be doing exactly what the managers of employment in large corporations are doing for each applicant before jobs are assigned. Such applicants are fitted to the positions they are suited to fill, and their salaries are based on their percentages of efficiency.

The average of human efficiency in the United States today is given at 51 per cent. This means that if our efficiency in health is that low, the 51 per cent. man has done no more work at 5 o'clock than the 100 per cent. man has at 1 o'clock, and while doing his work has not had the advantages of personality, personal appearance, good health, optimism, faith, honesty, poise, confidence, mental decision, courage, enthusiasm and ability—all of which a 100 per cent. man would have used in his work and profited much thereby. However, the 100 per cent. man was not born that way, and may have been a 51 per cent. man at one time. First of all he recognized his imperfections, and through system set about reconstructing himself, all of which any dentist can do if he will. The only difficulty is to be brought face to face with a list of our liabilities. Having once recognized them, there are plenty of ways and means at hand for self-improvement.

Study the questions carefully, and diagnose your own case before you answer.

Answer the questions frankly and accurately. There is a reason for every question, and each one is an important factor in your success or failure as a dentist.

Instead of answering "Yes" or "No," rate "Yes" as 100 and "No" as 0. If there is an intermediate stage rate you answer at a percentage that corresponds with your degree of self-assurance. Then add up the column of percentages and divide by 32. The answer will be your approximate grade in efficiency.

| | Answer in Percentage |
|---|---|
| 1. Do you like dentistry? | .......... |
| 2. Have you learned the most advanced methods of doing it? | .......... |
| 3. Are you working for the class of patients you want to work for? | .......... |
| 4. Is your income equal to that of the average business man of your acquaintance? | .......... |
| 5. Is your office equipped to the last degree of efficiency? | .......... |
| 6. Are you in perfect physical health? | .......... |
| 7. Is your personal appearance and that of your assistants beyond criticism? | .......... |
| 8. Do you wish your competitors well and never criticize them? | .......... |
| 9. Have you learned how to be optimistic under all circumstances? | .......... |
| 10. Have you eliminated pain from your work? | .......... |

| | Answer in Percentage |
|---|---|
| 11. Are you running your office on the same principles that a business man runs his on? | .......... |
| 12. What percentage of the fees you charge do you collect? | .......... |
| 13. What percentage of your patients become enthusiastic? | .......... |
| 14. What percentage of your associates are your patients? | .......... |
| 15. What percentage of pyorrhea do you diagnose before the pus stage? | .......... |
| 16. What percentage of pyorrhea do you cure? | .......... |
| 17. What percentage of follow-up prophylactic work do you do? | .......... |
| 18. Do you know what it costs you to run your business? | .......... |
| 19. Do you sell service to your patients rather than time and material? | .......... |
| 20. Do you make a careful and honest diagnosis of every case? | .......... |
| 21. Have you mastered a selling talk that convinces your prospective patient of the importance of dentistry in health preservation? | .......... |
| 22. Do you discuss price before rather than after an operation? | .......... |
| 23. Have you any successful system for utilizing time which would otherwise be lost? | .......... |
| 24. Do you investigate the status, financial and otherwise, of your prospective patients? | .......... |
| 25. Are you thought of in your community as a professional man rather than a mechanic? | .......... |
| 26. Do you insist on doing all the work necessary in your patients' mouths? | .......... |
| 27. Have you eliminated from your practice all work that can not be done at a profit (except clinic patients and justifiable charity work)? | .......... |
| 28. Are you saving a satisfactory amount of money each month? | .......... |
| 29. Are you discounting your supply bills? | .......... |
| 30. Are your co-workers striving to make your work a success? | .......... |
| 31. Do you use suggestion in every branch of your business? | .......... |
| 32. Are you constantly trying to improve your methods, both of doing dentistry and doing business? | .......... |
| Total | .......... |
| Divide total by 32 | .......... |

—*American Dentist.*

